# Development of a robust BH3 drug toolkit for precision medicine in hematologic malignancies

**DOI:** 10.7150/thno.107852

**Published:** 2025-04-21

**Authors:** Valentin Jacquier, Andréa Romero, Caroline Molinaro, Ritu Somayaji, Matthieu Abouladze, Ouissem Karmous Gadacha, Sara Ovejero, Hugues de Boussac, Ludovic Gabellier, Matthew S. Davids, Jérôme Moreaux, Charles Herbaux

**Affiliations:** 1 Institute of Human Genetics, UMR CNRS-Univ. Montpellier, 9002 Montpellier, France.; 2 Department of Clinical Hematology, CHU Montpellier, Montpellier, France.; 3 Department of Medical Oncology, Dana-Farber Cancer Institute, Boston, MA, USA.; 4 Department of Biological Hematology, CHU Montpellier, Montpellier, France.; 5 Diag2Tec, Montpellier, France.; 6 University of Montpellier, UFR Medicine, Montpellier, France.; 7 Institut Universitaire de France (IUF), Paris, France.

## Abstract

**Rationale:** In the era of precision medicine, there is a growing need for rapid reliable *ex vivo* functional assays capable of predicting treatment efficacy. One drug class that may particularly benefit from such assays is BH3 mimetics. These small molecules antagonize anti-apoptotic proteins such as BCL-2, MCL-1, or BCL-XL, on which cancer cells depend for their survival. A functional assay known as BH3 profiling was previously developed to measure those dependencies through the use of specific BH3-only peptides. A variation of this technique, dynamic BH3 profiling (DBP), allows for measuring changes in those dependencies, after *ex vivo* treatment with a drug of interest. Though well-validated to predict clinical response in hematologic malignancies, BH3 profiling technique requires the use of specialized BH3-only peptides and requires significant optimization to achieve reproducible results.

**Methods:** We used a toolkit of BH3 mimetics drugs as probes instead of BH3-only peptides. This technique reduces the complexity and cost by using Annexin V/7AAD staining instead of cytochrome c release as a functional readout for apoptosis. We also used cell lines as internal controls for a representative response to BH3 mimetics that allow us to easily compare and stratify patients according to their profile.

**Results:** We demonstrate that our new protocol enables apoptotic dependencies to be measured efficiently across different hematologic malignancies. In addition to a detailed description of the assay, we describe the results in several models including cell lines and primary tumor cells, both at baseline and dynamically after *ex vivo* drug treatments. We also compared BH3 toolkit baseline results on cell lines with those obtained using conventional BH3 profiling.

**Conclusion:** Overall, our data validates this streamlined BH3 drug toolkit, allowing for a more extensive use of the BH3 profiling technique.

## Introduction

One cancer hallmark that allows for cell proliferation and chemoresistance is the deregulation of apoptosis 1,2. Even though they display internal dysregulation, such as DNA damage or metabolic impairment, cancer cells can evade apoptosis through different mechanisms; one is the disruption of the balance between the different members of the *B Cell Lymphoma 2* (BCL2) protein family 3.

Despite sharing one or several *BCL-2 Homology* (BH) 3 domains, the members of the BCL-2 family exert different functions in the intrinsic pathway of apoptosis. They can be divided into three subgroups: the pro-apoptotic BH3-only proteins, which include BIM, BID, PUMA, BAD, MS1 and HRK; the anti-apoptotic proteins, represented by BCL-2, BCL-XL, BCL-W, MCL-1, BFL-1; and finally the pro-apoptotic effector proteins, which include BAX, BAK and BOK 3,4. After activation by the pro-apoptotic proteins, the effector protein can oligomerize and create pores in the mitochondrial outer membrane, therefore allowing the release of cytochrome c 5. The latter will activate APAF-1 and form the apoptosome, initiating the caspase cascade and *in fine* cell death 6. This process is regulated by anti-apoptotic proteins, which directly inhibit apoptosis by sequestrating activated pro-apoptotic proteins on one hand. On the other hand, they indirectly inhibit apoptosis by sequestering sensitizer BH3-only proteins 7. Therefore, the balance between the expression level and the activity of all BCL-2 family members determines whether or not a cell is primed for apoptosis.

To measure this level of priming, a technique called BH3 profiling was previously developed 8. Based on the discovery that BH3 domains of pro-apoptotic proteins can inhibit specific anti-apoptotic proteins 9,10, they first exposed isolated mitochondria to BH3 peptides and measured cytochrome c release by ELISA or Western Blot as an output for apoptosis triggering 11-13. Large numbers of cells are required for this technique. The next step was then to work on whole cell extract. However, delivering BH3 peptides directly to the mitochondria is necessary without damaging it. To this end, they used small amounts of detergent, such as digitonin, to permeabilize the plasma membrane without damaging mitochondria 14. It allowed internal antibodies to directly target cytochrome c and measure its release by flow cytometry. With these improvements, the BH3 profile of different subpopulations of a healthy tissue or tumor can be assessed using suitable markers.

Another version of this technique, dynamic BH3 profiling (DBP), has been developed and consists of measuring changes in mitochondrial priming upon *ex* vivo drug treatment 15. With this protocol, it is possible to measure early drug-induced changes in pro-apoptotic signaling and to identify modifications of anti-apoptotic dependencies. Recently, this technique has been coupled with microfluidics 16 or even microscopy 17 for a more precise or large-scale analysis.

With the emergence of a new type of drug class called BH3 mimetics, we believe that a new improvement in the BH3 profiling technique is possible. These small molecules mimic the effects of BH3 sensitizer protein by competing for binding in the anti-apoptotic protein catalytic pocket, which is why they are also used, among BH3-only peptides, in the BH3 profiling protocol. In addition, their small size allows them to pass through the plasma membrane. We use this property to shorten the protocol of BH3 profiling by removing the permeabilization step. Moreover, this also allows us to work on live cells to more closely mimic their normal physiology. The second notable change is the systematic use of 3 internal controls, each with a known dependency on BCL-2, BCL-XL, and MCL-1. We aim to improve reproducibility and comparability between assays and thus reduce variability linked to different analysis batches (time points, operators, etc.). Finally, the recent success of venetoclax in monotherapy or combinations in treating chronic lymphocytic leukemia (CLL), mantle cell lymphoma (MCL), or acute myeloid leukemia (AML), has led to the development of several BH3 mimetics, either specific or with several targets 18. Based on this variety of BH3 mimetics, we developed a new BH3 “toolkit” profiling protocol with internal cellular control that allows for a rapid and precise evaluation of anti-apoptotic dependencies in both hematologic malignancy cell lines and primary samples.

## Materials and methods

### Control cell line culture and cryopreservation

OCI-Ly1 (diffuse large B-cell lymphoma) and HEL (acute myeloid leukemia) cell lines were purchased from DSMZ (Braunschweig, Germany). JJN3 (multiple myeloma) was kindly provided by Dr. Van Riet (Bruxelles, Belgium). HEL and JJN3 cells were grown in RPMI-1640 Glutamax medium (61870044, Gibco, NY, USA), supplemented with 10% fetal bovine serum (FBS) (S1810-500, Dutscher, France). OCI-Ly1 cells were cultured in IMDM Glutamax (11504556, Gibco), supplemented with 10% FBS. Cultures were maintained at 37 °C in a humidified atmosphere with 5% CO_2_. Cells were frozen 48 h after passage, and their viability was checked by Annexin V/7AAD staining (640930, Biolegend, CA, USA) to ensure maximum viability. Each batch of cell lines vials was tested at least twice to certify their viability and BH3 mimetics response before being used as a control.

### Primary cells culture

Primary samples were obtained at the University Hospital of Montpellier after patients' written informed consent following the Declaration of Helsinki and the agreement of the Montpellier University Hospital Centre for Biological Resources (DC-2008-417). Primary AML samples were cultured for 24 h in RPMI-1640 Glutamax medium (61870044, Gibco) supplemented with human FLT-3 Ligand (50 ng/mL, PHC9414, Gibco), human SCF (50 ng/mL, PHC2116, Gibco), IL-3 (10 ng/mL, PHC0034, Gibco) and IL-6 (10 ng/mL, 200-06, Preprotech, MA, USA). For diffuse large B-cell lymphoma (DLBCL) primary samples, cells were cultured in RPMI-1640 Glutamax medium supplemented with CD40 ligand-His tag (10 ng/mL, 2706-CL, R&D systems, MN, USA), anti-histidine antibody (1 µg/mL, MAB050, R&D systems), IL-4 (50 pg/mL, 204-IL, R&D systems), IL-7 (1 ng/mL, 207-IL, R&D systems) and IL-10 (1 ng/mL, 217-IL, R&D systems). For mantle-cell lymphoma, RPMI-1640 Glutamax was supplemented with CD40 ligand-His tag and anti-histidine at the same concentration as previously described. Finally, multiple myeloma (MM) primary cells were cultured in RPMI-1640 Glutamax supplemented with IL-6 (10 ng/mL). For prolonged primary cell culture (more than 24 h), we used Resto-6 stromal cells as a cytokine provider. Resto-6 cells were plated in 96 well plates and cultured for 24 h in RPMI-1640 Glutamax medium before the addition of primary sample at a concentration of 4000 cells/mL.

### Conventional BH3 profiling

For baseline conventional BH3 profiling, 3 million cells of immortalized cell line were suspended in 1.65 mL MEB2P buffer (150 mM mannitol, 10 mM HEPES-KOH pH 7.5, 150 mM KCl, 1 mM EGTA, 1 mM EDTA, 0.1% BSA, 5 mM succinate, 0.25% poloxamer 188) prior to transferring 15 µL of the cell suspension with an automated multi-channel pipette to the BH3 profiling 384-well plate, which consist of 0.002% digitonin for cell membrane permeabilization, increasing concentrations of BIM, BAD, PUMA, HRK and MS1 11-mer pro-apoptotic peptides (New England Peptide, MA, USA) as well as BH3 mimetic ABT199 (venetoclax). Cells were incubated for 1 h in this plate, followed by fixation with 15 µL of 4% paraformaldehyde for 30 min and then neutralization with 15 µL of N2 buffer (1.7 M Tris, 1.25 M Glycine pH 9.1) for 20 min. Cells were then stained with 10 µL of a staining cocktail consisting of Alexa-488-conjugated cytochrome c (#612308, Biolegend) antibody and Hoechst 33342 (H3570, Invitrogen, MA, USA) and incubated overnight prior to analysis on the BD FACS Fortessa flow cytometry. Induction/increase in cytochrome c release, which indicates specific dependencies for anti-apoptotic protein, by HRK is measured for BCL-XL, MS1 for MCL-1, BAD for BCL-2 and/or BCL-XL and venetoclax for BCL-2. BIM was used as overall mitochondrial priming for apoptosis.

### BH3 drug toolkit

**Figure 1:**
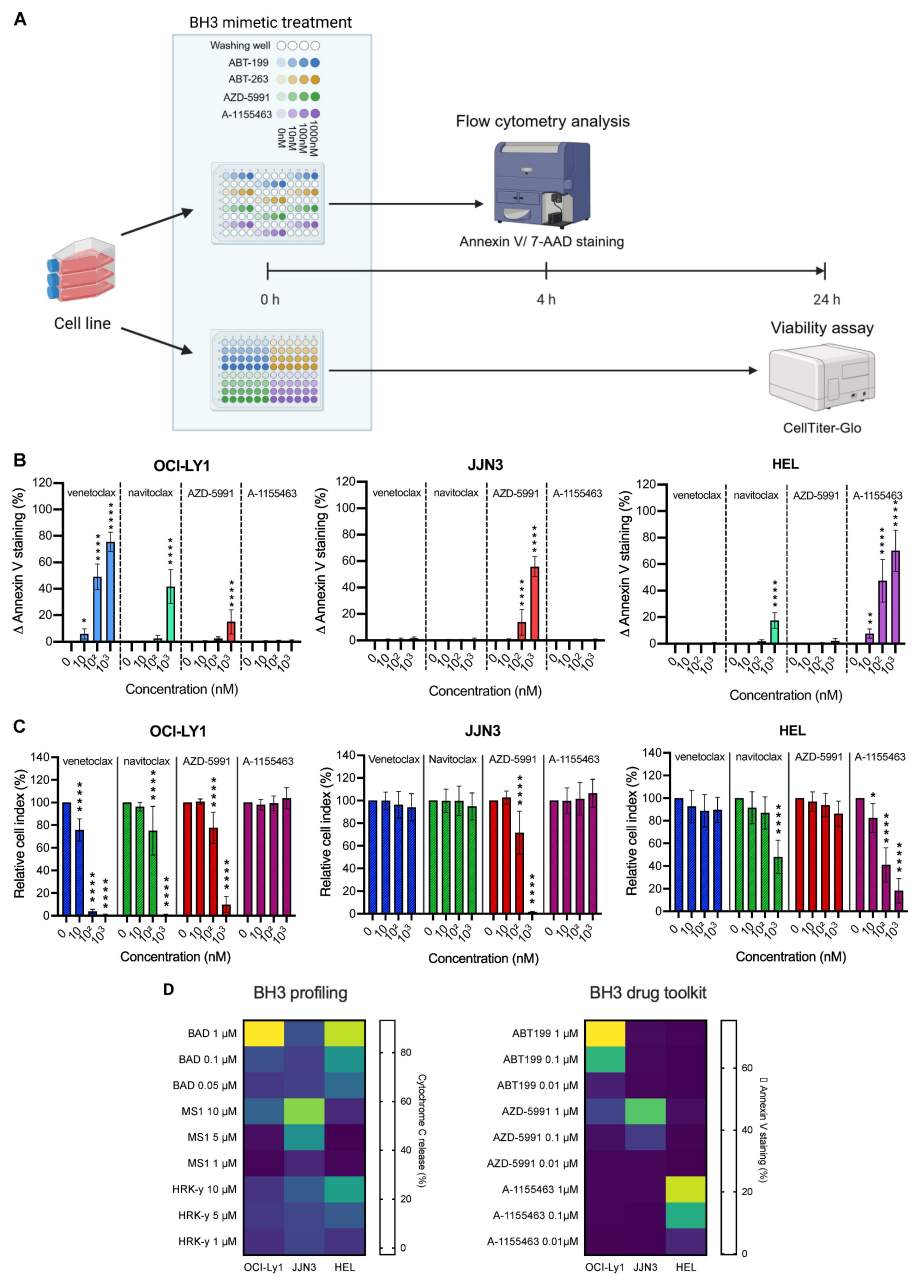
** Baseline BH3 drug toolkit on cell lines.** A) Diagram of the baseline BH3 toolkit protocol. Created with BioRender.com. B) Quantification of ∆ Annexin V after 4 h treatment with the BH3 toolkit (in percentage). Values are represented as the difference between the treated and control condition for each drug. The percentage of cells Annexin V+ only, are taken into account. C) Cell viability assessment by CellTiter-Glo® luminescent cell viability assay of BH3 toolkit on cell lines after 24 h treatment. Results were normalized to the cell density of the control condition for each BH3 mimetic in each cell line. D) Comparison between canonical baseline BH3 profiling (left panel) and BH3 toolkit protocol (right panel) of cell lines, depicted by a heatmap of cytochrome c loss intensity or ∆ Annexin V staining respectively, following individual BH3 peptides or BH3 mimetics incubations. All results are expressed as the mean ±SEM. of at least three biologically independent replicates. *p ≤ 0.05, ** p ≤ 0.01, *** p ≤ 0.001, **** p ≤ 0.0001.

For baseline protocol, primary tumor cells are thawed and immediately plated in two 96-well plates (167008, ThermoFisher Scientific, MA, USA) at 20,000 cells/well concentration using an automatic liquid handler (EPmotion 5070, Eppendorf, Germany). Cells are then treated with 10 nM, 100 nM, 1 µM of either venetoclax (ABT-199, Selleckchem S8048, Selleckchem, TX, USA), navitoclax (ABT-263, Selleckchem S1001), AZD-5991 (Selleckchem, S8643) or A-1155463 (Selleckchem, E2926) and incubated for 4 h (Figure 1A). Control cells (OCI-Ly1, JJN3, and HEL) are thawed and seeded in the same 96-well plates at a concentration of 0.5 million cells/mL for OCI-Ly1 and JJN3, or 0.3 million cells/mL for HEL. Control cells are then treated with 1 µM of venetoclax, navitoclax, AZD-5991, or A-1155463 and incubated for 4 h. Cells were then stained with 7AAD and Annexin V-APC detection kit (640930, Biolegend, CA, USA) following the manufacturer's protocol, and 5 µL of precision counting beads (424902, Biolegend) were added just before analysis with a BD LSRFortessa Flow cytometer (BD Bioscience, NJ, USA). The other plate is processed following the same protocol 24 h later. Data was processed using Kaluza Software (Beckman, CA, USA), and cell numbers were determined by counting beads and cell counts. Cell toxicity was monitored by comparing cell numbers between the 24 h and 48 h plates.

For Dynamic BH3 profiling, 100 µL of primary cell suspensions containing either dimethylsulfoxide or a drug of interest were added to two 96-well plates and incubated for 20 h. Finally, we added 50 µL of the previously described BH3 mimetics for a final concentration of 10 nM, 100 nM, and 1 µM, and plates were incubated for an additional 4 h. For the first plate, cells were stained and analyzed using the same protocol as previously described. The second plate was stained and analyzed as previously described 24 h later.

For each BH3 mimetics, the difference of annexin V staining between the treated and the control condition was calculated and represented as ∆ Annexin V for each concentration for primary cells and control cells. Scores for each anti-apoptotic protein dependency (BCL-2, MCL-1, and BCL-XL) were then calculated as the ratio between the ∆ Annexin V of the primary sample and the ∆ Annexin V of the specific control cell line at 1 µM (OCI-Ly1 for BCL-2, JJN3 for MCL-1 and HEL for BCL-XL). Radar charts were generated using Excel 2024 (Microsoft Corporation, WA, USA).

### Cytotoxicity assay

For cell lines, cell cytotoxicity was assessed 24 h after flow cytometry analysis to ensure early apoptotic events resulted in cell death. Cells were cultured in a 96-well plate for 48 h with the same concentration of BH3 mimetics as previously described. Cell viability was evaluated using CellTiter-Glo (CTG) Luminescent Assay (G7573, Promega, WI, USA) according to the manufacturer's protocol, and luminescence was measured using a Centro LB 960 luminometer (Berthold Technologies, Bad Wildbad, Germany). Cytotoxicity was then evaluated as the difference between the treated and the control condition.

### Statistical analysis

All the results were expressed as the mean ± SEM of at least three biologically independent replicates. Every condition was compared to the control condition using a two-way ANOVA test and marked as statistically significant (*) when the p-value was lower than 0.05. GraphPad Prism 10 (Insight Partners, NY, USA) was used to perform statistical analyses and represent the results.

## Results

### The new BH3 drug toolkit protocol can detect specific anti-apoptotic dependencies

To facilitate the BH3 profiling protocol, we use BH3 mimetics instead of peptides, thus avoiding the need for permeabilization steps and allowing us to work on living cells. We have built up a BH3 mimetic toolkit consisting of venetoclax, AZD-5991, and A-1155463, which are specifically inhibiting BCL-2, MCL-1, and BCL-XL, respectively, and navitoclax that inhibits BCL-2, BCL-XL, and BCL-w (Figure 1A). As the cells are not permeabilized, we could not use intra-cellular antibody to stain cytochrome c in order to detect the early onset of apoptosis. Instead, we use Annexin V and 7AAD staining (Figure 1A). Additionally, to translate apoptotic events triggered solely by BH3 mimetics, we express the percentage of Annexin V positive cells as the difference between the treated condition and its control, which we call ∆ Annexin V. Finally, our gating strategy (Supplementary Figure 1A) focuses on living cells and annexin V only positive cells so that only early apoptotic events are reported. Indeed, late apoptotic events may not be caused by BH3 mimetics treatment directly or can be due to cell membrane integrity loss, which make them not specific of anti-apoptotic dependencies.

We first tested our protocol on cell lines with known apoptotic dependencies: OCI-Ly1 (DLBCL) for BCL-2 19, JJN3 for MCL-1 20, and HEL for BCL-XL 21. Consistent with those data, we detected a significant increase in ∆ Annexin V with all doses of venetoclax in OCI-Ly1 cell line (+6.0% (P = 0.0122) at 10 nM, + 49.0% at 100 nM and + 75.4% at 1 µM (p < 0.0001)) (Figure 1B). Those results were corroborated by the induction of apoptosis with navitoclax treatment at the maximum dose (+ 41.7% ∆ Annexin V, (p < 0.0001)) but not with A-1155463 treatment. Surprisingly, we also detected a small but significant increase in ∆ Annexin V with the MCL-1 inhibitor, with an increase of 15.0% (p < 0.0001). In the JJN3 cell line, only the MCL-1 inhibitor could strongly induce early apoptotic events, as shown by the increase of Annexin V staining by 55.4% (p < 0.0001). Finally, we observed the most substantial increase in Annexin V staining with BCL-XL inhibitors in the HEL cell line, starting at 7.6% ∆ Annexin V at only 10 nM of A-1155463 and even reaching 69.1% ∆ Annexin V (p < 0.0001) at 1 µM. With navitoclax, the increase was only 17.9% (p < 0.0001) at maximum dosage, in line with the known lower specificity of this drug.

To confirm that early apoptotic events translate into cell death, we performed a viability assay using CTG. Consistently, we obtain the most substantial decrease in cell viability with 1 µM of venetoclax for OCI-Ly1 (- 99.3% (p < 0.0001)), 1 µM of AZD-5991 for JJN3 (- 98.3% (p < 0.0001)) and 1 µM of A-1155463 for HEL (- 81.9% (p < 0.0001)) (Figure 1C). We also observed decreased cell viability with the intermediate doses, corresponding to the previously measured variation ∆ Annexin V. Hence, we wanted to assess if there is a correlation relationship between the two parameters. We performed a correlation analysis (Supplementary Figure 1B), and we obtained a strong positive correlation with a Pearson correlation coefficient of 0.8918 (p < 0.0001). Moreover, our model can predict the amount of cell death with a high reliance (R^2^ = 0.7953).

Finally, to support the robustness of our approach, we compared it with the “traditional” BH3 profiling technique. We treated our cell lines with BAD BH3 peptide, which inhibits BCL-2, BCL-XL, and BCL-w, or MS1 and HRK-y peptides, which inhibit MCL-1 and BCL-XL respectively. Consistently with our expectations, we obtained a comparable BH3 profile with the two techniques. BAD peptide induced strong cytochrome c release in OCI-Ly1 and HEL cell lines (93.2% and 83.9% respectfully (p < 0.0001)) and MS1 peptide induced apoptosis in 76.1% of the cells in the JJN3 cell line (p < 0.0001) (Figure 1D). Of note, the HRK-y peptide induced only a mild release of cytochrome c in the HEL cell line at 50 µM (50.5% (p < 0.0001)) and another MCL-1 inhibitor, S63845, failed to induce a significant release of cytochrome c in JJN3 cells, even at the highest dose (11.6% P = 0.9724) (Supplementary Figure 1C). Finally, venetoclax and another BCL-XL inhibitor, A-133, induced strong cytochrome c release in OCI-Ly1 (96.3% (p < 0.0001)) and HEL (90.4% (p < 0.0001)) cell lines, respectively.

### BH3 toolkit can measure changes in apoptotic dependencies upon treatment in cell lines

**Figure 2:**
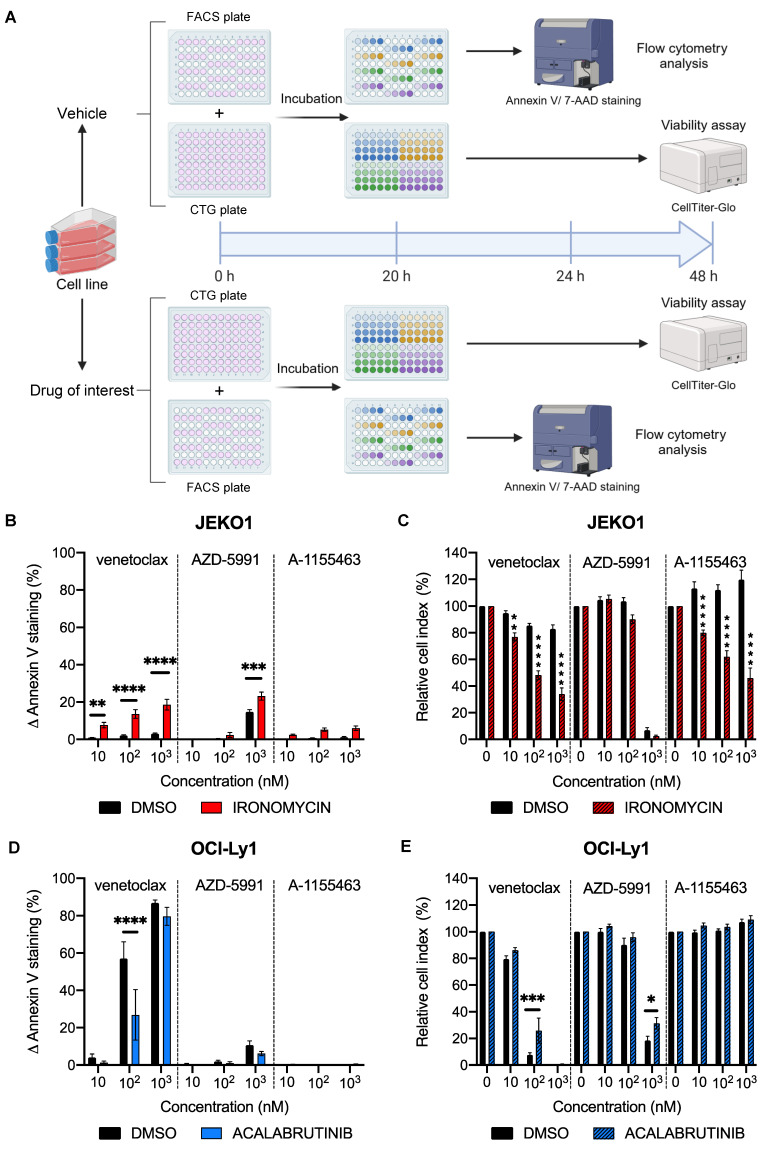
** Dynamic BH3 toolkit profiling on cell lines.** A) Diagram of the dynamic BH3 toolkit protocol. Created with BioRender.com. B) and D) Detection of changes in anti-apoptotic dependencies upon treatment with either the vehicle or the drug of interest. Cells were treated for 20 h with ironomycin before staining with Annexin V and 7AAD. ∆ Annexin V is represented as the difference between treated and untreated for each dose of BH3 mimetics, for vehicle and pre-treated conditions. C) and E) Cell viability assessment by CellTiter-Glo® luminescent cell viability assay of BH3 toolkit on JEKO1 and OCI-Ly1 cells after 48 h treatment for the vehicle and treated conditions. Results were normalized to the cell density of the control condition for each BH3 mimetic in the vehicle and pre-treated condition. All results are expressed as the mean ±SEM. of at least three biologically independent replicates. *p ≤ 0.05, ** p ≤ 0.01, *** p ≤ 0.001, **** p ≤ 0.0001.

As we confirmed that our protocol can detect specific apoptotic dependencies at the basal level, we wanted to evaluate it in a dynamic context. We added an incubation period of 20 h with either a vehicle (DMSO) or a drug of interest (Figure 2A). Cells were then treated for 4 h with the BH3 toolkit, resulting in a 24 h total incubation time for the BH3 profiling plate and 48 h for the cell viability plate. Four plates per experiment were used, one pair corresponding to the control condition and the other to the tested condition. First, we tested our protocol in the context of mantle cell lymphoma (MCL). Indeed, previous work from our team 22 showed that iron chelator ironomycin can induce apoptosis in lymphoma B cells. Given the efficiency of another iron chelator, deferasirox, in MCL 23,24, we wanted to test ironomycin in this pathology and its potential effect on apoptosis. After 24 h of treatment, we measured an increase by 9.9% (P = 0.0007) in ∆ Annexin V with an MCL-1 inhibitor (Figure 2B) in the MCL cell line JEKO1. Interestingly, we measured a significant increase in Annexin V staining with all doses of the BCL-2 inhibitor in the treated condition but not in the control (+ 6.5% (P = 0.0496), + 12.1% (p < 0.0001) and + 16.9% (p < 0.0001) for 10, 10^2^, 10^3^ nM respectively). Once again, we wanted to confirm that these changes in apoptotic dependencies could translate into changes in cell death upon treatment. We thus measured a larger decrease in cell viability in the treated condition with venetoclax and A-1155436. Indeed, in the venetoclax-treated condition, cell viability is only 34.0% with ironomycin, whereas it is 82.3% with the vehicle (Figure 2C). Interestingly, with the BCL-XL inhibitor, the difference between the treated and control conditions reached 73.7% (p < 0.0001), underlining the onset of new apoptotic dependencies with this treatment. With the MCL1 inhibitor, we were not able to detect significant differences between the vehicle and treated condition, as both conditions are close to 100% cell death (6.9% and 2.7% respectively (P = 0.9968)).

To evaluate the robustness of our protocol, we next tested it in another context. We treated OCI-Ly1 cells with acalabrutinib, a selective Bruton tyrosine kinase inhibitor that is evaluated in the treatment of DLBCL 25. Surprisingly, we observed a significant reduction in BCL-2 dependency with 100 nM of venetoclax, as we observed a decrease of 31.9% (p < 0.0001) of ∆ Annexin V staining (Figure 2D). This translates into a significant (P = 0.0006) augmentation of cell viability with acalabrutinib (40.6%) compared to the control (Figure 2E). With these results, we demonstrated that our protocol could detect changes in apoptotic dependencies in cell lines.

### Optimisation of BH3 drug toolkit protocol on primary cells with internal control

**Figure 3:**
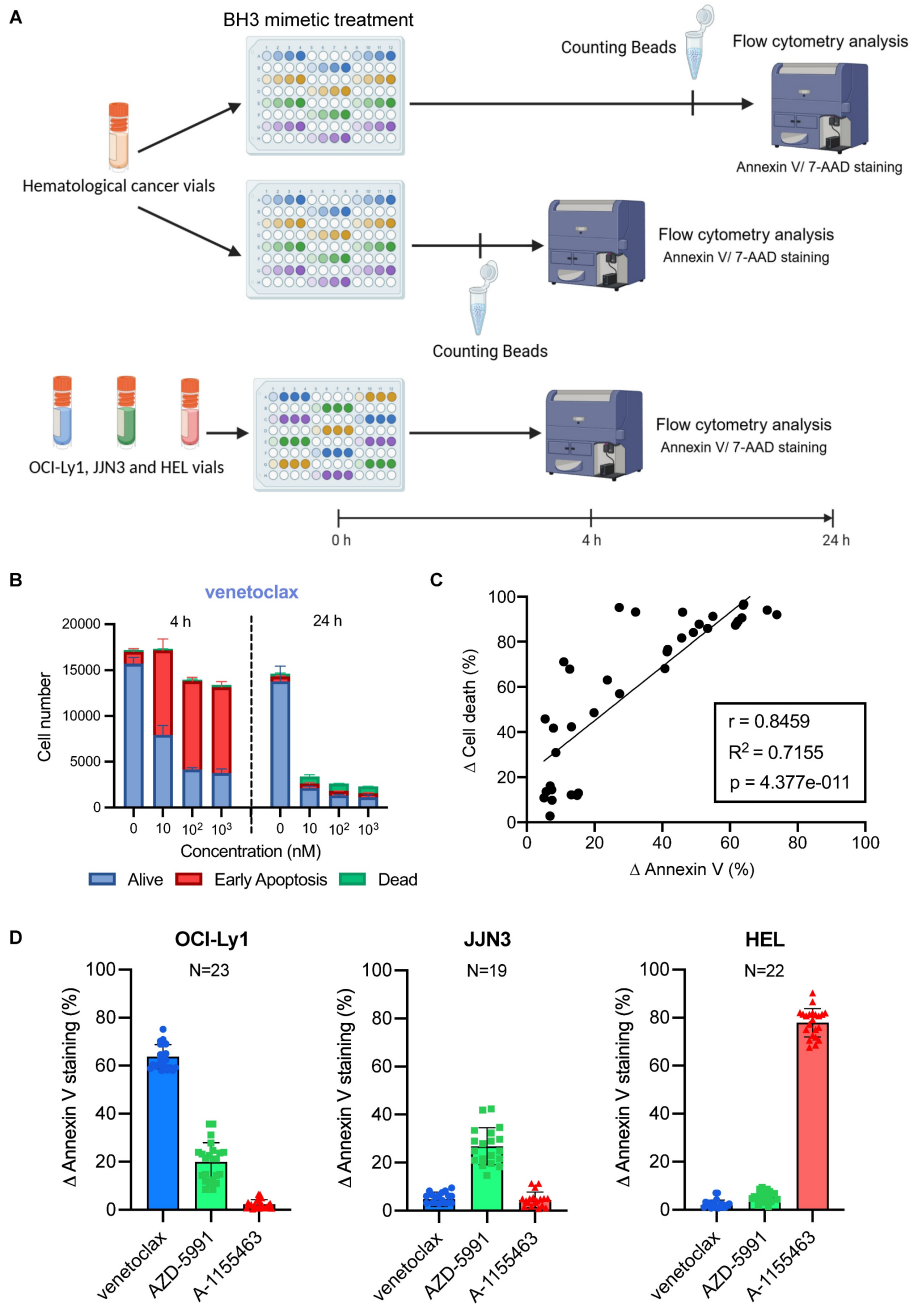
** Baseline BH3 toolkit profiling parameters.** A) Diagram of the baseline BH3 toolkit protocol for primary samples. Created with BioRender.com. B) Relative cell number was calculated with fluorescent beads of CLL primary cells treated with venetoclax for 4 h. Number of cells are represented as 'Alive' (double negative), 'Early Apoptosis' (Annexin V only positive cells) and 'Dead' (7AAD positive and double positive cells). Only living cells (Supplementary Figure 1A) are represented. C) Correlation analysis of ∆ Annexin V and ∆ Cell death of primary cells. ∆ Cell death is the difference between the percentage of viable cells in the control and the treated condition. n = 4 primary samples. D) ∆ Annexin V of OCI-Ly1, JJN3 and HEL cells upon thawing in response to 1 µM. 'n' represents the number of independent experiments with a new vial each time.

We next applied our assay to the evaluation of primary tumor cells. Due to the limited proliferation and metabolic activity of primary cells *in vitro,* CTG or other metabolism-based proliferation assays do not provide reliable results. Hence, we replaced the cell proliferation plate with another Annexin V/7AAD plate (Figure 3A), where we measured cell number by adding fluorescent counting beads in the 4 h and 24 h plates.

We first tested this protocol on CLL primary samples known for their significant BCL-2 dependency. As expected, we detected a substantial increase in Annexin V staining with each dose of venetoclax (Figure 3B), which translated into a drop of living cell number 24 h later (17 153 living cells with 9 218 of them being in early apoptosis cells at 4 h against 2607 living cells with 486 of them being in early apoptosis at 24 h, for 10 nM of venetoclax). The number of control cells remains stable (17 000 cells at 4 h and 14 281 cells at 24 h), with a slight decrease between the two time points (possibly due to the effect of thawing). In contrast, the BCL-XL inhibitor failed to induce a significant ∆ Annexin V (3.6%). This translates into a uniform number of cells between the doses and the different time points (Supplementary Figure 2A). To further confirm that fluorescent counting beads allow to monitor cell state during the experiment, we performed a correlation analysis between the differences in cell number between the control and the tested condition called ∆ Cell death and the ∆ Annexin V (Figure 3C). Both parameters correlated significantly (Pearson coefficient r = 0.8459 p < 0.0001), and the early apoptotic events detected at 4 h enabled us to precisely predict the percentage of cell death 20 h later (R^2^ = 0.71).

In order to normalize experiments performed by different people at various times, and compare different primary samples from patients, we used cellular internal control, representative of each anti-apoptotic dependency as standards. Therefore, we used the same cell lines described above (OCI-Ly1 representative of BCL-2 dependency, JJN3 for MCL-1, and HEL for BCL-XL). We initially identified the BH3 mimetic concentration that could induce the most significant and consistent triggering of apoptosis across the three cell lines. We needed to reduce the number of tests for concentration to fit all the control cell lines on one plate.

During testing, 1 µM stands out as the concentration with the lowest standard deviation across all cell lines (Supplementary Figure 2B). Then, we chose to work with cells thawed from viably frozen vials to ensure that we could perform the BH3 drug profiling toolkit whenever needed, as we wanted to eliminate the need for cell culture and its inherent challenges (passages, insufficient number of cells, contamination, etc.). Thus, we first tested the reproducibility of our control response to 1 µM of BH3 mimetics upon thawing (Figure 3D). We obtained consistent responses for all three controls, with a standard deviation (SD) of 5.0 for venetoclax with OCI-Ly1, 7.7 for AZD-5991 with JJN3, and 5.9 for A-1155463 with HEL. We noticed that the JJN3 cell line has a lower Δ Annexin V than the other two cell lines. We tested two additional cell lines with known MCL-1 anti-apoptotic dependencies: OCI-AML3 26 and LP-1 27 (Supplementary Figure 2C). We observed similar ranges of Δ Annexin V, but with lower consistency and reduced cell viability after thawing. Thus, we decided to keep JJN3 as our MCL-1 control. We also compared AZD-5991 with S63845, another MCL-1 inhibitor (Supplementary Figure 2D). This compound did not result in a significant increase in Annexin V staining compared to AZD-5991. This was confirmed through classical BH3 profiling (Supplementary Figure 1C). To confirm that variability was reduced to a minimum, we tested the mean response of different batches of vials (Supplementary Figure 2E), and we highlighted that most of the variability came from inter-batch variation rather than intra-batch. Based on these results, we tested the response of at least two vials of each batch of controls to ensure that their response falls into the previously determined range, before using them in our BH3 drug profiling toolkit primary cells protocol. The last parameter we had to check was the consistency of response between fresh and frozen samples to support our choice to work with cells thawed from viably frozen vials for our controls. We observed some small differences with all cell lines, with a mean difference of 11.5% for OCI-Ly1, 7.5% for JJN3 and 6.6% for HEL (Supplementary Figure 3A). We observed the same small variability with primary cells (Supplementary Figure 3B), with patient 1 and patient 2 having a mean difference between fresh and frozen sample of 4.7% and 4.5% respectively. Even for low mitochondrial priming cells such as patient 3's one, the mean difference between fresh and frozen sample was only of 5.5%. With those parameters in check, we started to profile different patient samples with various hematologic malignancies.

### The BH3 drug toolkit identified heterogeneous dependencies among different hematologic malignancies

**Figure 4:**
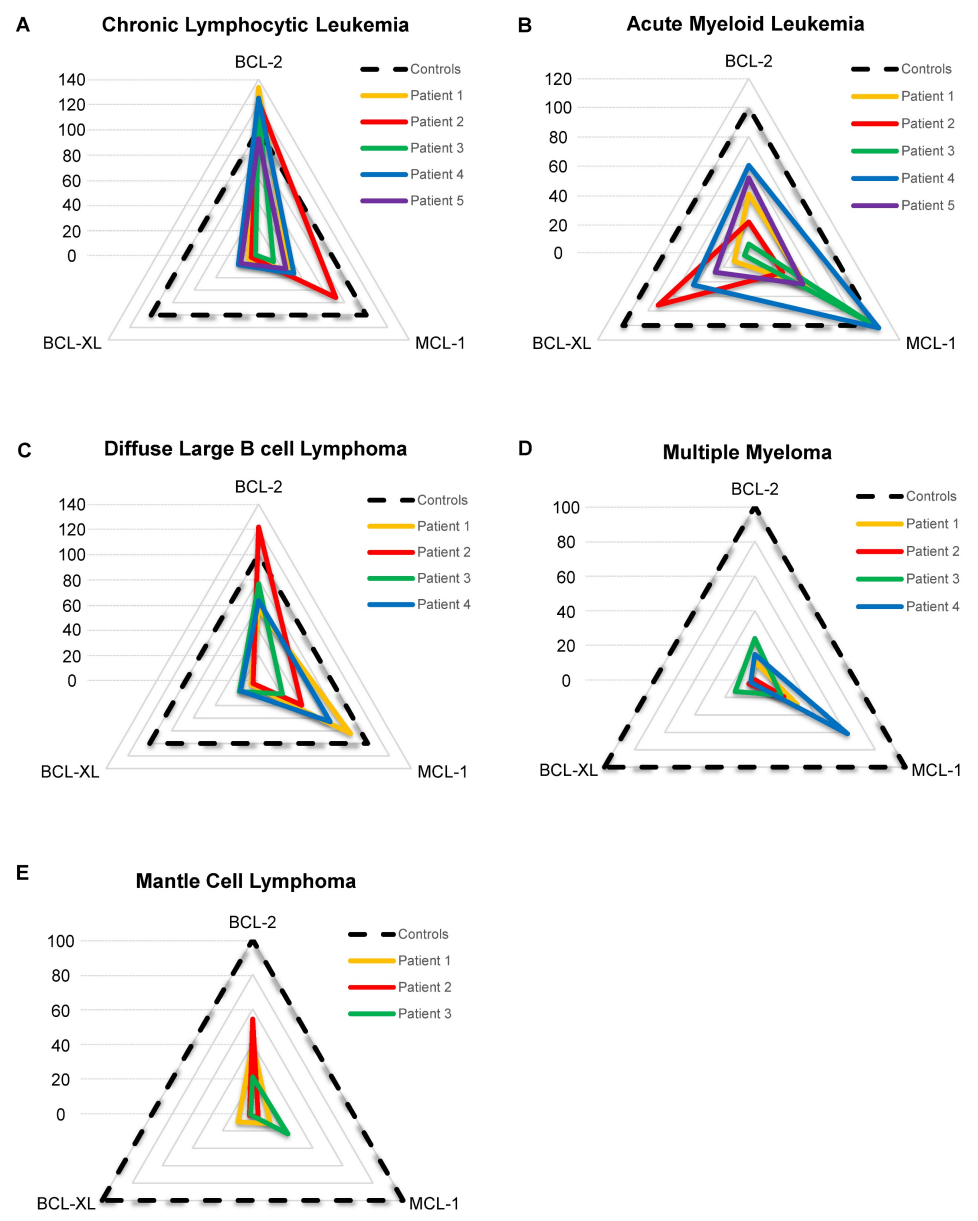
** BH3 profiling of different hematological malignancies samples.** A-E) Radar chart representation of the BCL-2, MCL-1 and BCL-XL scores for each patient grouped by hematological malignancies. The dotted line represents control response as OCI-Ly1 for BCL-2, JJN3 for MCL-1 and HEL for BCL-XL.

To make results easier to read and to standardize them, we developed a score that represents cells' dependencies relative to each internal control. The score is based on the primary sample response to the BH3 toolkit, divided by the corresponding control response (Supplementary Material 1). OCI-Ly1, JJN3 and HEL were used as control for the dependency to BCL-2, MCL-1 and BCL-XL respectively. We weighed the MCL-1 score by 30 since JJN3 response to its given BH3 mimetic is the lowest to provide a homogeneous representation. Then, the different scores are plotted on a radar chart, which makes a rapid and easy reading of a patient's BH3 drug profile (Figure 4A-E). For cells from patients with CLL, we observed that the different samples are relatively homogeneous, with 5 primary samples having a very high BCL-2 score (ranging from 92.0 to 138.6), consistent with the literature 28. This transcribes a very high BCL-2 dependency, with a higher response than the control BCL-2 cell line OCI-Ly1. Moreover, all patients have a mild MCL-1 score (mean score is 34.6) and a low BCL-XL score (mean score is 11.0), except for patient 2, having a MCL-1 score of 71.7. For AML patients (Figure 4B), scores were heterogeneous, with some patients being strictly MCL-1 dependent (patient 3) and some others showing a strong BCL-XL dependency (patient 2, BCL-XL score = 71.9). The other 3 patients displayed mild BCL-2 and MCL-1 dependency, with patient 4 having the highest ones. For diffuse large B cell lymphoma (DLBCL) patients (figure 4C), all of them have a mild to high BCL-2 score, with patient 2 reaching 121,8. Moreover, patients 1, 2 and 4 also have a mild to high MCL-1 scores ranging from 39.5 to 84.3. For multiple myeloma (MM) samples (Figure 4D), shown previously to be primarily MCL-1 dependent 29, all patients' highest score was the MCL-1 one, with patient 4 reaching a score of 61,58. However, the first 3 patients' highest scores were 28.3, 19.3 and 24, respectively, showing low anti-apoptotic dependencies for these primary samples. In the same way, MCL samples we tested (Figure 4E) had low anti-apoptotic dependencies. Nonetheless, the first 2 patients had mild BCL-2 dependency, with scores of 40.6 and 54.3, respectively. Thus, by using various primary samples from different pathologies, we demonstrated that our protocol allows for the measurement of specific anti-apoptotic dependencies of primary samples, and the stratification of patients.

### The BH3 toolkit allows dynamic evaluation of primary tumor cells

**Figure 5:**
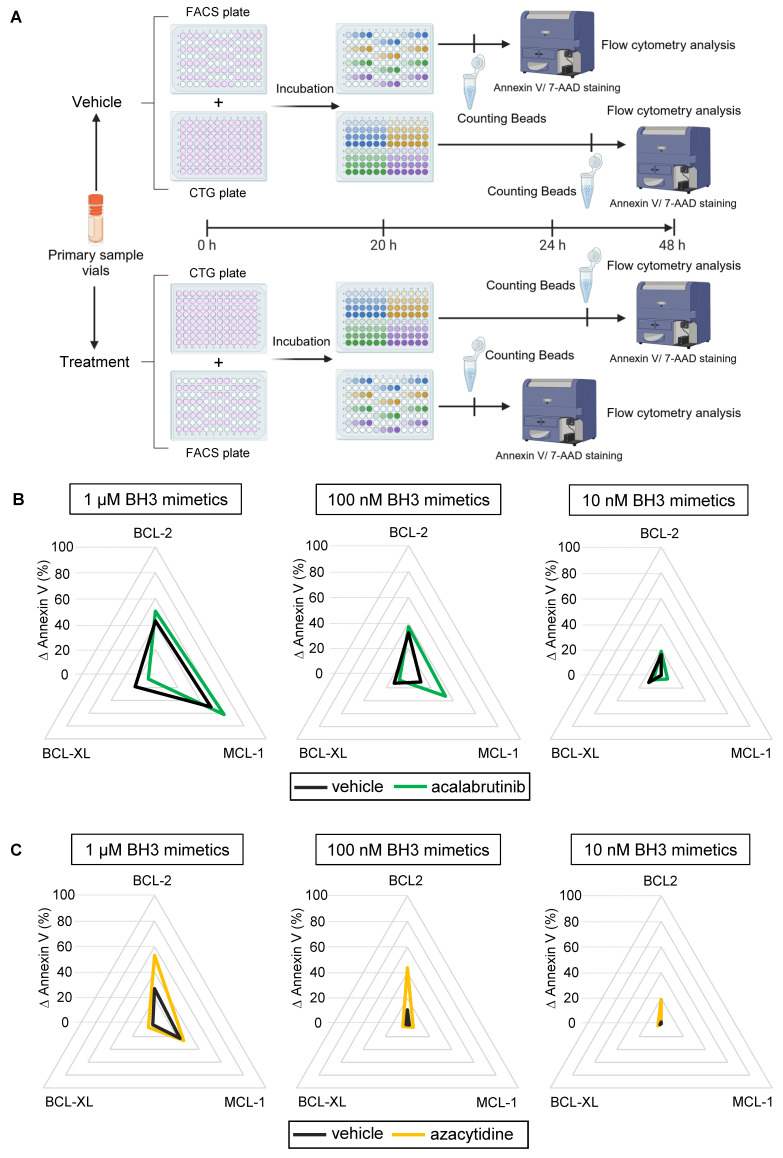
** Dynamic BH3 profiling of primary samples.** A) Diagram of the dynamic BH3 toolkit protocol on primary samples. Created with BioRender.com. B) Radar chart representation of the different ∆ Annexin V for 1 µM (left panel) 100 nM (middle panel) and 10 nM (right panel) of the BH3 toolkit on CLL primary sample treated with the vehicle (DMSO, black line) or acalabrutinib (green line) during 24 h. C) Radar chart representation of the different ∆ Annexin V for 1 µM (left panel), 100 nM (middle panel) and 10 nM (right panel) of the BH3 toolkit on AML primary sample treated with the vehicle (DMSO, black line) or azacytidine (yellow line) during 24 h.

Finally, we evaluated our protocol in primary tumor cells within a dynamic context of *ex vivo* drug treatment (Figure 5A). As the canonical dynamic BH3 profiling, this technique can identify pro-apoptotic drug combinations in a specific disease or patient. To test this protocol, we treated patient primary samples with an appropriate targeted therapy and our BH3 toolkit to see if changes in apoptotic dependencies could be detected. One variation with the baseline protocol is that we removed the control plate, since the BH3 drug profile of the treated condition is compared to the vehicle one, thus improving the workflow. We first incubated CLL primary cells with 1 µM of acalabrutinib, which is indicated in the treatment of this disease. This treatment slightly increased BCL-2 and MCL-1 dependencies at 1 µM of our BH3 toolkit (+ 7.6% and + 11,9% ∆ Annexin V respectively), probably because cells already reached their maximum mitochondrial priming (Figure 5B). Surprisingly, it decreased BCL-XL dependency with a 11.9% reduction of ∆ Annexin V. Nevertheless, with submicromolar dose, we measured an even more important increase in ∆ Annexin V with MCL-1 inhibitor (+ 21.9% at 100 nM). Likewise, in a second set of experiments, we tested our protocol using azacytidine, which is a hypomethylating agent approved for the treatment of AML in combination with venetoclax. Here, we observed that 1 µM of azacytidine for 24 h significantly increased BCL-2 dependency at every dose administered, with an increase in ∆ annexin V of 25,9% at 1 µM, 32.83% at 100 nM and 17.6% at 10 nM of venetoclax (Figure 5C). Interestingly, we could not detect any apoptosis onset at 10 nM of BH3 toolkit in the control condition. This confirms that our BH3 profiling toolkit allows for detecting either increases or changes in apoptotic dependencies of patient samples.

We then aimed to generate preliminary data to evaluate the feasibility of the BH3 toolkit in predicting treatment response. To achieve this, we selected the setting of the combination of azacytidine and venetoclax for treating AML in elderly subjects. We tested 8 AML patients' primary samples with baseline BH3 toolkit (Supplementary Figure 3C) and we observed various BH3 dependencies profiles, with some patients having mild BCL-2 and MCL-1 dependencies (Patient 3, 6, 7, and 8) or low BCL-2 and BCL-XL dependencies for patient 5. On the other hand, some patients are dependent on only one anti-apoptotic protein, MCL-1 for patient 1 and BCL-2 for patients 2 and 4. We also performed DBP using a treatment with azacytidine at 1 µM *ex vivo* (Supplementary Figure 3D). We observed increased dependency on BCL-2 in patient samples with a low dependency in baseline (like patients 2, 5, and 8). We collected clinical data from these patients, all of whom initially responded, which appears consistent with our biological data.

## Discussion

We demonstrate here that our novel protocol allows for detecting specific anti-apoptotic dependencies, both in cell lines and primary cells, in an accessible, quick and cost-efficient manner. Moreover, we have shown that it can detect changes in those dependencies upon exposure to a drug of interest. This simplification of the traditional BH3 profiling protocol has the potential to lead to a greater use of this type of functional technique. Indeed, BH3 profiling has already enabled the discovery of new therapeutic targets and drug combinations in solid tumors 30-32 and hematological malignancies 33-35. However, its cost and the difficulties of mastering it leave room to broaden its accessibility.

Compared to traditional BH3 profiling the first critical change we evaluated was to use BH3 mimetic drugs instead of BH3 peptides, leading to the obviation of the permeabilization steps, which can be a source of variability in results. Of note, depending on the supplier, it also led to a 7-fold reduction in costs. Other teams have shown that a toolkit of BH3 mimetics could be used to detect apoptotic dependencies 36 or guide the use of certain drugs in patients 37 but never directly compared it on this scale, with the use of BH3 peptides, and demonstrating similar activity of both types of compounds. We note that different types of BH3 mimetic drugs can be used to compose those toolkits, allowing to measure different anti-apoptotic dependencies based on their specificity at the same doses. A potential limitation of this technique is that there is no pan BCL-2 family inhibitor capable of measuring the overall priming of the cells, such as the BIM BH3-only peptide used in traditional BH3 profiling. We used navitoclax to counteract this problem, but it can only inhibit BCL-2, BCL-XL, and BCL-w, not taking into account cells with either MCL-1, BFL-1 or other anti-apoptotic dependencies. This problem could be overcome by using combinations of BH3 mimetics, but this would also mean increasing the number of conditions tested, increasing the complexity of the protocol. This limitation could be addressed in the future through the development of BH3 mimetics capable of inhibiting several anti-apoptotic proteins that could mimic the BIM BH3 peptide.

Another second major improvement with our novel protocol is our use of Annexin V and 7AAD staining as the marker for early apoptosis instead of cytochrome c release measurement that is used in the “canonical” protocol. Indeed, the significant advantages of Annexin V staining are its ease and speed of use, the elimination of the permeabilization steps, as well as its relatively limited cost, although one could argue that it is not the earliest apoptotic event, as cytochrome c release is. We also noticed that ∆ Annexin V under 5% failed to be significantly different from the untreated condition (Figure 2B) but still translated into cell death 24 h later. This underlines a sensitivity issue that could be due to different apoptosis induction times among cell types 38,39 but may not be sufficient for every cell type or treatment. To counteract this, a real-time Annexin V assay could be used, as it would allow for the detection of earlier or later apoptotic events, adding a time parameter to mitochondrial priming that could be modified by a given drug and detectable in DBP. Another limitation with Annexin V staining is its lack of fixability. It requires performing experiments immediately, which can be logistically challenging, especially with fresh patient samples, as one cannot control the experiment start time. The emergence of fixable apoptosis and viability dye could overcome this problem, but those products usually need additional washing steps, which are a source of variability and could induce cell loss with aspiration steps.

Cells state during the experiment should be closely monitored among all sources of variability. Indeed, we did find slight differences in responses between fresh and frozen samples (Supplementary Figure 2C and 2D). Two possible explanations for this include that fresh samples tend to have better overall viability, allowing measurement of apoptotic priming of a larger amplitude as it is calculated as the difference between the treated and control priming state, or that sample handling can influence sensitivity to BH3 mimetics and other cell parameters, as previously suggested 40. Moreover, thawing can lead to abnormal apoptosis triggered by artificial cleavage of caspases 41. Hence, working with fresh samples seems preferable, but the results obtained with frozen samples appear comparable. Moreover, working with frozen cells allows for more flexibility and sample availability. We also conducted preliminary assays to evaluate the feasibility of the BH3 toolkit (baseline and DBP) in predicting treatment response to the combination of azacytidine and venetoclax in AML. Our results appear promising and suggest the need for a dedicated study to assess the predictive power of our assay in this and other contexts.

We presented a protocol designed for hematological malignancies, as they are non-adherent cells, facilitating the workflow for flow cytometry. However, one of the assets of our protocol is that it is highly adaptable. Therefore, only minor modifications would be necessary for the protocol to be applied to solid tumors. Adherent cells could be cultured, then treated with the BH3 drug toolkit, detached with trypsin-EDTA, and dispatched in an Annexin V/7AAD containing plate with our layout, while the CTG plate does not need to be adapted. The cellular internal controls are other parameters that can be adapted depending on the laboratory needs and means. Indeed, they can be replaced with cell lines available in the facility after being tested for dependency and consistency with the BH3 drug toolkit.

In conclusion, our streamlined, reproducible protocol has the potential to broaden accessibility to BH3 profiling to allow more investigators to utilize this functional precision medicine technique to further understand the deregulation of apoptosis in cancer and how to overcome it therapeutically.

## Supplementary Material

Supplementary figures and material.

## Abbreviations

AML: acute myeloid leukemia
BCL-2: b-cell lymphoma 2
CTG: CellTiter-Glo®
CLL: chronic lymphocytic leukemia
DBP: dynamic BH3 profiling
DLBCL: diffuse large b-cell lymphoma
MCL: mantle cell lymphoma
MM: multiple myeloma

## References

[1] Hanahan D, Weinberg RA (2011). Hallmarks of cancer: the next generation. Cell.

[2] Plati J, Bucur O, Khosravi-Far R (2008). Dysregulation of apoptotic signaling in cancer: molecular mechanisms and therapeutic opportunities. J Cell Biochem.

[3] Youle RJ, Strasser A (2008). The BCL-2 protein family: opposing activities that mediate cell death. Nat Rev Mol Cell Biol.

[4] Wolf P, Schoeniger A, Edlich F (2022). Pro-apoptotic complexes of BAX and BAK on the outer mitochondrial membrane. Biochim Biophys Acta Mol Cell Res.

[5] Korsmeyer SJ, Wei MC, Saito M, Weiler S, Oh KJ, Schlesinger PH (2000). Pro-apoptotic cascade activates BID, which oligomerizes BAK or BAX into pores that result in the release of cytochrome c. Cell Death Differ.

[6] Fan T-J, Han L-H, Cong R-S, Liang J (2005). Caspase family proteases and apoptosis. Acta Biochim Biophys Sin.

[7] Kale J, Osterlund EJ, Andrews DW (2018). BCL-2 family proteins: changing partners in the dance towards death. Cell Death Differ.

[8] Certo M, Del Gaizo Moore V, Nishino M, Wei G, Korsmeyer S, Armstrong SA (2006). Mitochondria primed by death signals determine cellular addiction to antiapoptotic BCL-2 family members. Cancer Cell.

[9] Chen L, Willis SN, Wei A, Smith BJ, Fletcher JI, Hinds MG (2005). Differential targeting of prosurvival Bcl-2 proteins by their BH3-only ligands allows complementary apoptotic function. Mol Cell.

[10] Kim H, Rafiuddin-Shah M, Tu H-C, Jeffers JR, Zambetti GP, Hsieh JJ-D (2006). Hierarchical regulation of mitochondrion-dependent apoptosis by BCL-2 subfamilies. Nat Cell Biol.

[11] Deng J, Carlson N, Takeyama K, Dal Cin P, Shipp M, Letai A (2007). BH3 profiling identifies three distinct classes of apoptotic blocks to predict response to ABT-737 and conventional chemotherapeutic agents. Cancer Cell.

[12] Del Gaizo Moore V, Schlis KD, Sallan SE, Armstrong SA, Letai A (2008). BCL-2 dependence and ABT-737 sensitivity in acute lymphoblastic leukemia. Blood.

[13] Del Gaizo Moore V, Brown JR, Certo M, Love TM, Novina CD, Letai A (2007). Chronic lymphocytic leukemia requires BCL2 to sequester prodeath BIM, explaining sensitivity to BCL2 antagonist ABT-737. J Clin Invest.

[14] Ryan JA, Brunelle JK, Letai A (2010). Heightened mitochondrial priming is the basis for apoptotic hypersensitivity of CD4+ CD8+ thymocytes. Proc Natl Acad Sci U S A.

[15] Pallis M, Burrows F, Ryan J, Grundy M, Seedhouse C, Abdul-Aziz A (2017). Complementary dynamic BH3 profiles predict co-operativity between the multi-kinase inhibitor TG02 and the BH3 mimetic ABT-199 in acute myeloid leukaemia cells. Oncotarget.

[16] Manzano-Muñoz A, Yeste J, Ortega MA, Martín F, López A, Rosell J (2022). Microfluidic-based dynamic BH3 profiling predicts anticancer treatment efficacy. NPJ Precis Oncol.

[17] Bhola PD, Ahmed E, Guerriero JL, Sicinska E, Su E, Lavrova E (2020). High-throughput dynamic BH3 profiling may quickly and accurately predict effective therapies in solid tumors. Sci Signal.

[18] Townsend PA, Kozhevnikova MV, Cexus ONF, Zamyatnin AA, Soond SM (2021). BH3-mimetics: recent developments in cancer therapy. J Exp Clin Cancer Res CR.

[19] Guièze R, Liu VM, Rosebrock D, Jourdain AA, Hernández-Sánchez M, Zurita AM (2019). Mitochondrial reprogramming underlies resistance to BCL-2 inhibition in lymphoid Malignancies. Cancer Cell.

[20] Flanagan L, O'Dwyer ME, Murphy P, Quinn J, Glavey S, Ni Chonghaile T (2021). Venetoclax and Epigenetic Modifiers: Promising Novel Combinations for the Treatment of Multiple Myeloma. Blood.

[21] Kuusanmäki H, Dufva O, Vähä-Koskela M, Leppä A-M, Huuhtanen J, Vänttinen I (2023). Erythroid/megakaryocytic differentiation confers BCL-XL dependency and venetoclax resistance in acute myeloid leukemia. Blood.

[22] Devin J, Cañeque T, Lin Y-L, Mondoulet L, Veyrune J-L, Abouladze M (2022). Targeting Cellular Iron Homeostasis with Ironomycin in Diffuse Large B-cell Lymphoma. Cancer Res.

[23] Samara A, Shapira S, Lubin I, Shpilberg O, Avigad S, Granot G (2021). Deferasirox induces cyclin D1 degradation and apoptosis in mantle cell lymphoma in a reactive oxygen species- and GSK3β-dependent mechanism. Br J Haematol.

[24] Vazana-Barad L, Granot G, Mor-Tzuntz R, Levi I, Dreyling M, Nathan I (2013). Mechanism of the antitumoral activity of deferasirox, an iron chelation agent, on mantle cell lymphoma. Leuk Lymphoma.

[25] Strati P, de Vos S, Ruan J, Maddocks KJ, Flowers CR, Rule S (2021). Acalabrutinib for treatment of diffuse large B-cell lymphoma: results from a phase Ib study. Haematologica.

[26] Wang Q, Hao S (2019). A-1210477, a selective MCL-1 inhibitor, overcomes ABT-737 resistance in AML. Oncol Lett.

[27] Quentmeier H, Geffers R, Hauer V, Nagel S, Pommerenke C, Uphoff CC (2022). Inhibition of MCL1 induces apoptosis in anaplastic large cell lymphoma and in primary effusion lymphoma. Sci Rep.

[28] Reyes A, Siddiqi T (2023). Targeting BCL2 pathways in CLL: a story of resistance and ingenuity. Cancer Drug Resist Alhambra Calif.

[29] Al-Odat OS, von Suskil M, Chitren RJ, Elbezanti WO, Srivastava SK, Budak-Alpddogan T (2021). Mcl-1 Inhibition: Managing Malignancy in Multiple Myeloma. Front Pharmacol.

[30] Singh R, Yu S, Osman M, Inde Z, Fraser C, Cleveland AH (2023). Radiotherapy-Induced Neurocognitive Impairment Is Driven by Heightened Apoptotic Priming in Early Life and Prevented by Blocking BAX. Cancer Res.

[31] Fitzgerald M-C, O'Halloran PJ, Kerrane SA, Ní Chonghaile T, Connolly NMC, Murphy BM (2023). The identification of BCL-XL and MCL-1 as key anti-apoptotic proteins in medulloblastoma that mediate distinct roles in chemotherapy resistance. Cell Death Dis.

[32] Potter DS, Du R, Bohl SR, Chow K-H, Ligon KL, Bueno R (2023). Dynamic BH3 profiling identifies pro-apoptotic drug combinations for the treatment of malignant pleural mesothelioma. Nat Commun.

[33] Herbaux C, Kornauth C, Poulain S, Chong SJF, Collins MC, Valentin R (2021). BH3 profiling identifies ruxolitinib as a promising partner for venetoclax to treat T-cell prolymphocytic leukemia. Blood.

[34] Zhu F, Crombie JL, Ni W, Hoang N-M, Garg S, Hackett L (2024). Hypomethylating agent decitabine sensitizes diffuse large B-cell lymphoma to venetoclax. Haematologica.

[35] Chong SJF, Zhu F, Dashevsky O, Mizuno R, Lai JX, Hackett L (2023). Hyperphosphorylation of BCL-2 family proteins underlies functional resistance to venetoclax in lymphoid malignancies. J Clin Invest.

[36] Butterworth M, Pettitt A, Varadarajan S, Cohen GM (2016). BH3 profiling and a toolkit of BH3-mimetic drugs predict anti-apoptotic dependence of cancer cells. Br J Cancer.

[37] Gomez-Bougie P, Maiga S, Tessoulin B, Bourcier J, Bonnet A, Rodriguez MS (2018). BH3-mimetic toolkit guides the respective use of BCL2 and MCL1 BH3-mimetics in myeloma treatment. Blood.

[38] Feng Y, Wu J, Feng X, Tao D, Hu J, Qin J (2007). Timing of apoptosis onset depends on cell cycle progression in peripheral blood lymphocytes and lymphocytic leukemia cells. Oncol Rep.

[39] Palchaudhuri R, Lambrecht MJ, Botham RC, Partlow KC, Van Ham TJ, Putt KS (2015). A Small Molecule that Induces Intrinsic Pathway Apoptosis with Unparalleled Speed. Cell Rep.

[40] Struyf N, Österroos A, Vesterlund M, Arnroth C, James T, Sunandar S (2024). Delineating functional and molecular impact of ex vivo sample handling in precision medicine. NPJ Precis Oncol.

[41] Schmidt-Mende J, Hellström-Lindberg E, Joseph B, Zhivotovsky B (2000). Freezing induces artificial cleavage of apoptosis-related proteins in human bone marrow cells. J Immunol Methods.

