# Supplementary material for: Development of a robust BH3 drug toolkit for precision medicine in hematologic malignancies
Source: Theranostics. 2025 Apr 21;15(12):5705–18. doi: 10.7150/thno.107852 (PMC12068295; doi:10.7150/thno.107852)
Supplement: Supplementary file 1 — Supplementary figures and material. [file thnov15p5705s1.pdf]

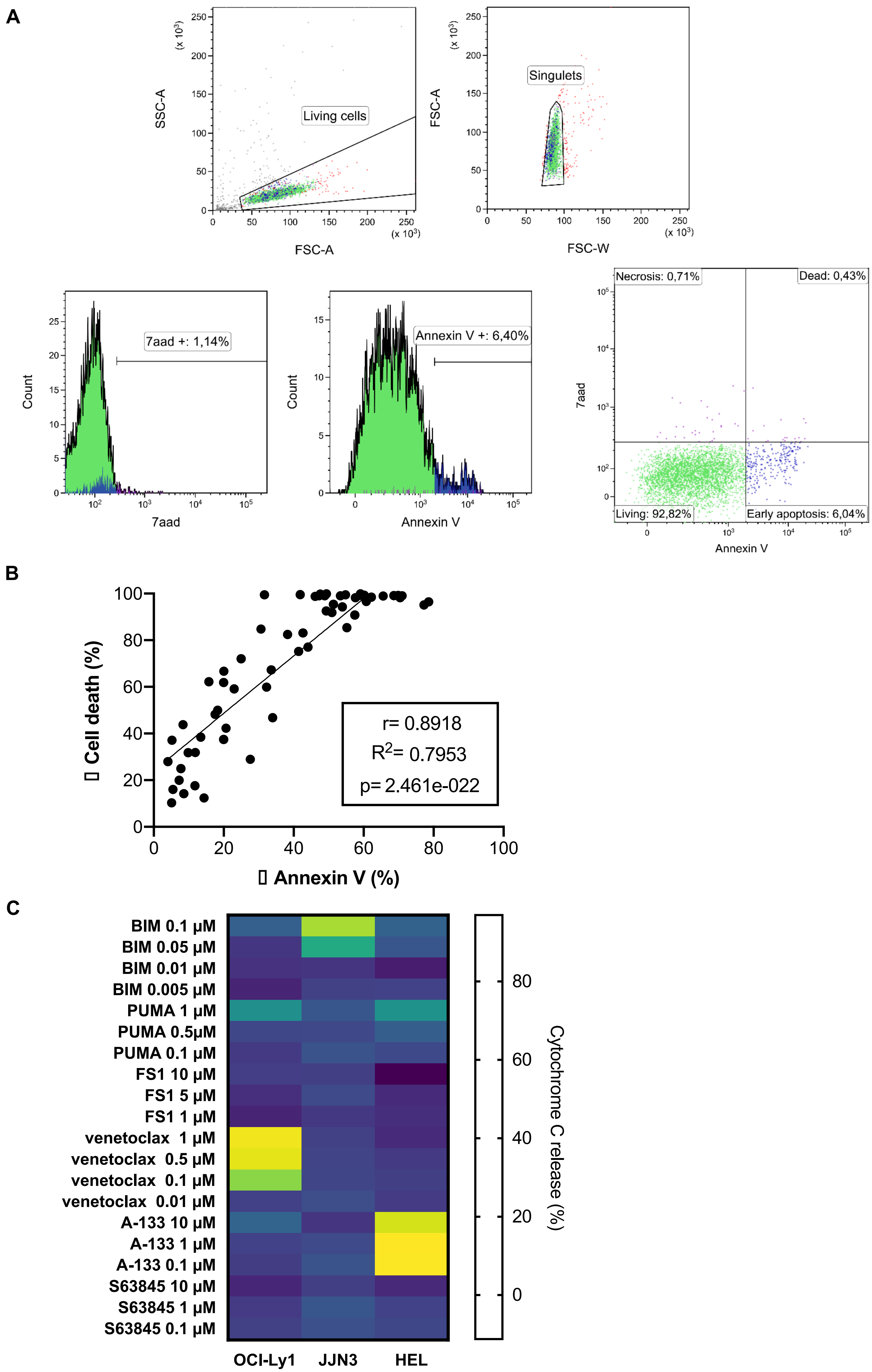
A
Living cells
SSC-A
FSC-A
Singulets
FSC-A
FSC-W
7aad +: 1,14%
Count
7aad
Annexin V +: 6,40%
Count
Annexin V
Necrosis: 0,71%
Dead: 0,43%
Living: 92,82%
Early apoptosis: 6,04%
7aad
Annexin V
B
▯ Cell death (%)
▯ Annexin V (%)
r= 0.8918
R2= 0.7953
p= 2.461e-022
C
BIM 0.1 µM
BIM 0.05 µM
BIM 0.01 µM
BIM 0.005 µM
PUMA 1 µM
PUMA 0.5µM
PUMA 0.1 µM
FS1 10 µM
FS1 5 µM
FS1 1 µM
venetoclax 1 µM
venetoclax 0.5 µM
venetoclax 0.1 µM
venetoclax 0.01 µM
A-133 10 µM
A-133 1 µM
A-133 0.1 µM
S63845 10 µM
S63845 1 µM
S63845 0.1 µM
OCI-Ly1
JJN3
HEL
Cytochrome C release (%)

Supplementary Figure 1:

A) Flow-cytometry gating strategy. From left to right and from up and down. FSC-A vs SSC-A allow us to identify living cells due to their granularity. FSC-A vs FSC-W allow us to select single cells. 7AAD histogram helps us confirm the gating strategy in the FSC-A vs SSC-A plot. Annexin V histogram allows for the detection of all Annexin V positive cells. 7AAD vs Annexin V plot allows for the discrimination between four cell states: double negative cells are alive, 7AAD-only positive cells are dead from necrosis, Annexin V-only positive cells are living cells in which apoptosis has been triggered, double positive cells are dead from either necrosis or apoptosis and are therefore only labeled as dead. B) Correlation analysis of Δ Annexin V and Δ Cell death on cell lines. Δ Cell death is the difference between the percentage of viable cells in the control and the treated condition. n = 26. C) Canonical baseline BH3 profiling of cell lines, depicted by a heatmap of cytochrome c loss intensity or Δ Annexin V following individual BH3 peptides or BH3 mimetics incubations.

Supplementary Figure 2

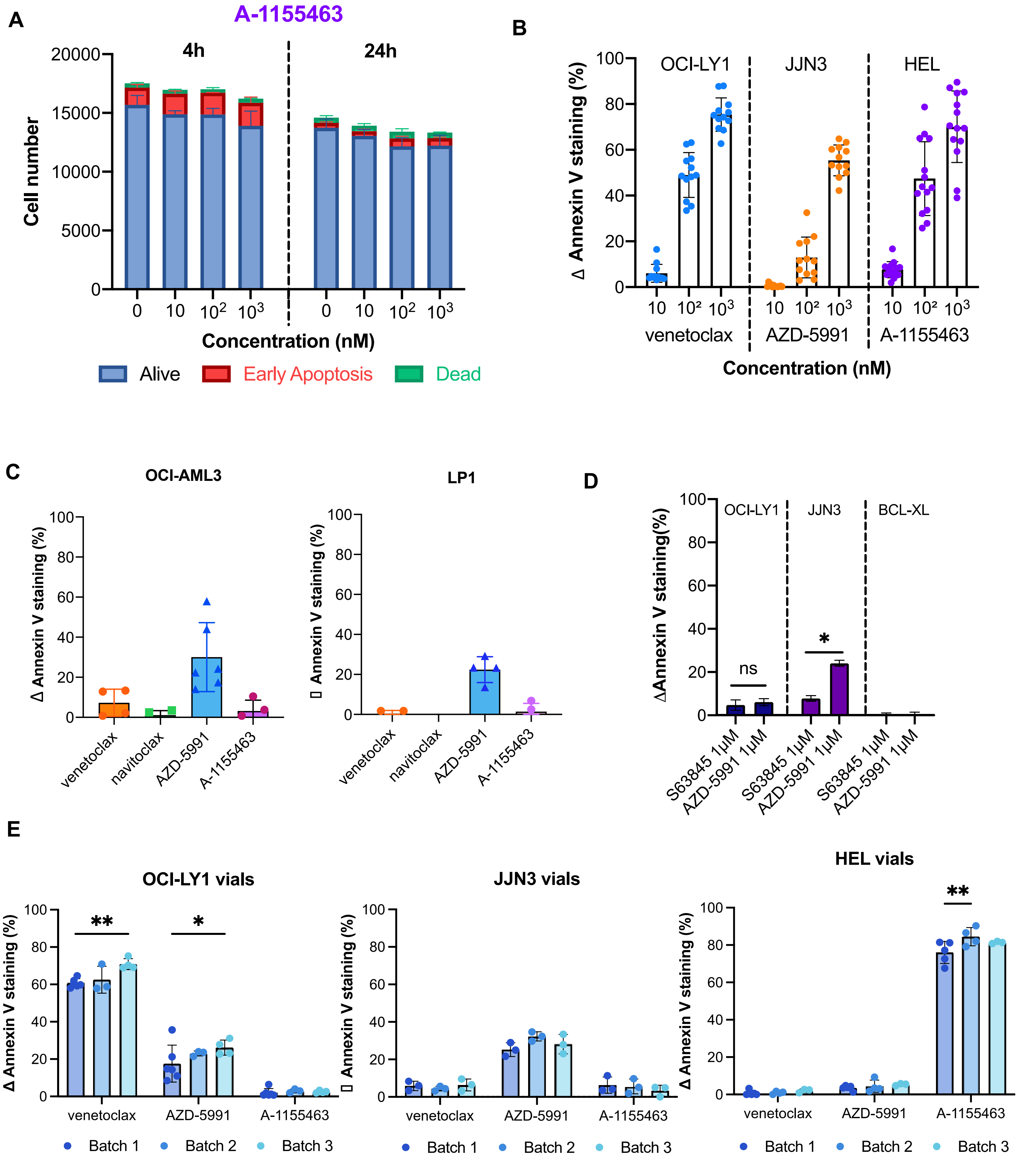

Supplementary Figure 2:

A) Relative cell number was calculated with fluorescent beads of CLL primary cells treated with A-1155463 for 4h. Number of cells are represented as 'Alive' (double negative), 'Early Apoptosis' (Annexin V only positive cells) and 'Dead' (7AAD positive and double positive cells). Only living cells (Supplementary Figure 1A) are represented. B) Measurement of mean response of OCI-LY1, JJN3 and HEL live cells to several doses of respectively venetoclax, AD-5991, A-1155463. Each dot represents an independent measurement C) Quantification of Δ Annexin V after 4h treatment with the BH3 toolkit (in percentage) of OCI-AML3 (left panel) and LP1 (right panel) cell lines. Values are represented as the difference between the treated and control condition for each drug. The percentage of cells AnnexinV+ only, are taken into account. Each dot represents an independent measurement D) Comparison between 1 µM treatment of control cell lines frozen vials with either S63845 or AZD-5991. E) Measurement of mean response to BH3 mimetics between different vials of control cell lines. Each dot represents one vial and each bar represents the mean response of all vials of the same batch.

All results are expressed as the mean ±SEM. of at least three biologically independent replicates. $*p \leq 0.05$, $** p \leq 0.01$, $*** p \leq 0.001$, $**** p \leq 0.0001$

Supplementary Figure 3

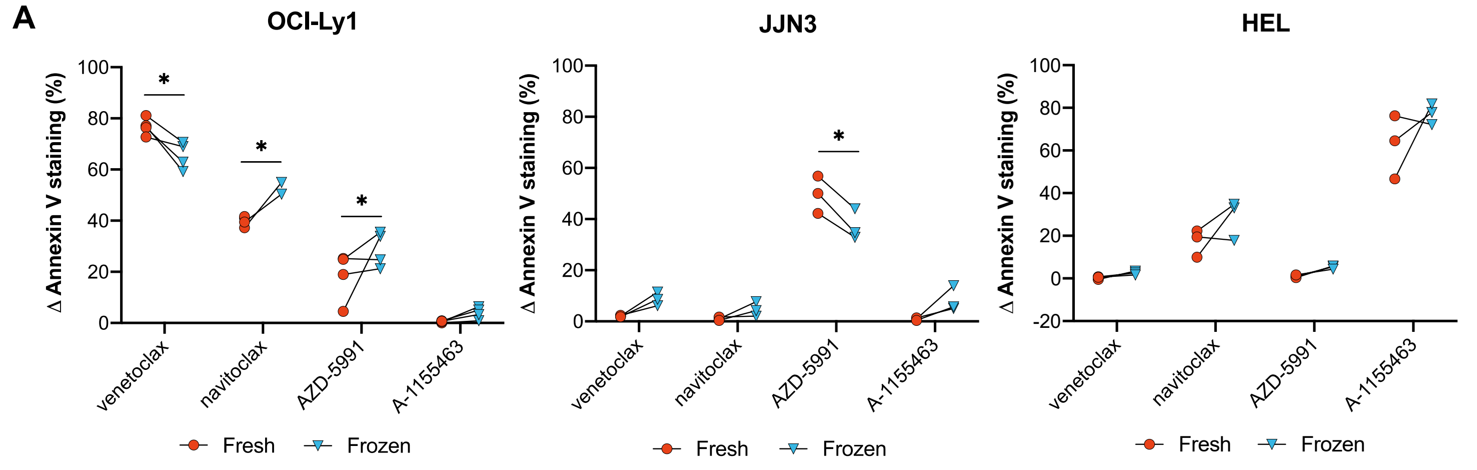

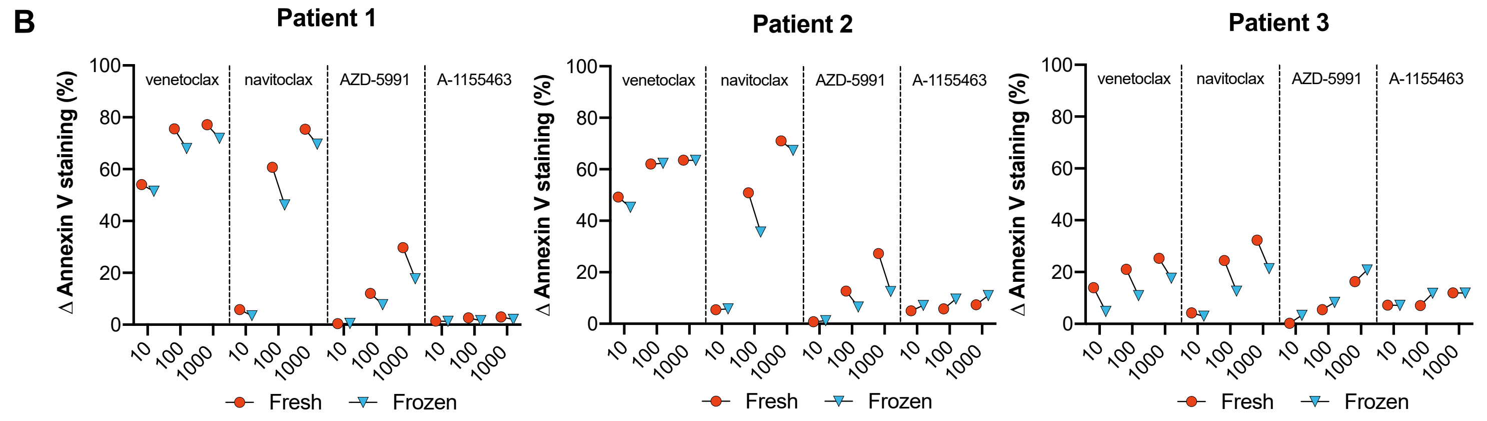

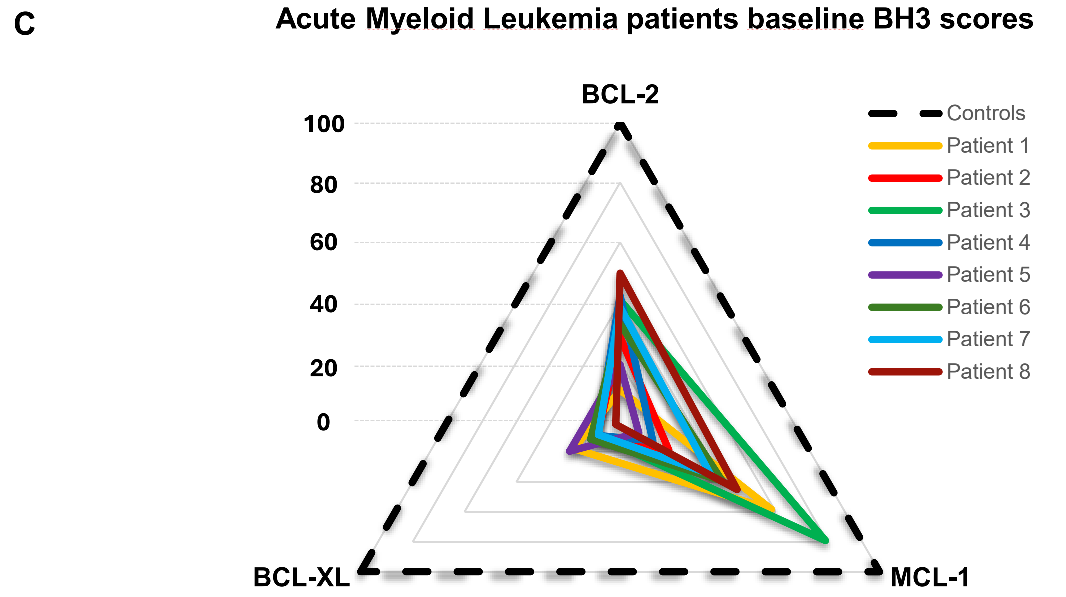

D

	Difference of Δannexin V between azacytidine and control conditions					
	BCL2			MCL1		
	10nm	100nm	1µm	10nm	100nm	1µm
Patient 1	1,57666667	0,308333	-1,74333	-1,66667	-18,74	-16,44
Patient 2	6,39333333	10,66	10,42	-1,61667	8,52	3,11
Patient 3	-3,1733333	-2,16	-4,52667	-1,44667	-2,67	-5,53
Patient 4	18,7	-0,19333	-3,94333	0,446667	1,38	3,25
Patient 5	6,59666667	9,75	8,06	-0,115	5,208333	6,73
Patient 6	-12,103333	9,943333	12,00333	-0,56167	3,828333	3,48
Patient 7	-0,2133333	1,433333	-1,73667	0,103333	-1,68333	-1,94667
Patient 8	17,64	32,83333	25,88667	0,115	3,483333	3,266667

Supplementary Figure 3:

A) Difference in mean Δ Annexin V staining between fresh and frozen samples of the same cells upon treatment with BH3 mimetics. B) Difference in mean Δ Annexin V staining between fresh and frozen sample of the same CLL patient upon BH3 mimetics treatment. C) Radar chart representation of the BCL-2, MCL-1 and BCL-XL scores for each patient. The dotted line represents control response as OCI-LY1 for BCL-2, JJN3 for MCL-1 and HEL for BCL-XL. D) Table representing differences in mean Δ Annexin V staining between azacytidine treated and control conditions of patients' samples presented in Supplementary Figure 3C.

All results are expressed as the mean ±SEM. of at least three biologically independent replicates. *$p \leq 0.05$, ** $p \leq 0.01$, *** $p \leq 0.001$, **** $p \leq 0.0001$

Supplementary Material 1:

$$\mathrm{BCL}-2\ \mathrm{score} = \frac{Primary\ sample\ response\ to\ 1\ \mu M\ venetoclax}{OCI{-}Ly1\ response\ to\ 1\ \mu M\ of\ venetoclax} \times 100$$

$$\mathrm{MCL}-1\ \mathrm{score} = \frac{Primary\ sample\ response\ to\ 1\ \mu M\ AZD{-}5991}{JJN3\ response\ to\ 1\ \mu M\ of\ AZD{-}5991 + 30} \times 100$$

$$\mathrm{BCL}-\mathrm{XL}\ \mathrm{score} = \frac{Primary\ sample\ response\ to\ 1\ \mu M\ A{-}1155463}{HEL\ response\ to\ 1\ \mu M\ of\ A{-}1155463} \times 100$$
